# Critical considerations for the use of deep learning models in clinical oncology prediction

**DOI:** 10.3389/fonc.2026.1730967

**Published:** 2026-02-24

**Authors:** Daren Zhao

**Affiliations:** Faculty of Social Sciences and Humanities, Mahidol University, Salaya, Nakhon Pathom, Thailand

**Keywords:** breast cancer, clinical prediction models, clinical research, deep learning models, generalizability

In a recent study on clinical prediction models, Liu (1) and colleagues developed a deep learning model to predict ALN pCR in ALN-positive breast cancer patients undergoing NAT by analyzing longitudinal ultrasound images of the primary tumor and ALNs. Their findings provide an evidence-based rationale for omitting unnecessary axillary lymph node dissection. This study serves as a representative case of clinical prediction modeling in oncology research, with its preliminary results indicating promising potential for clinical translation. However, it is imperative for us to acknowledge that a critical gap remains between the technical development of prediction models and their practical application in real-world clinical decision-making. We think that these challenges reflect common issues widely encountered by researchers in implementing clinical prediction models, which urgently require recognition and deep analysis.

First and foremost, comprehensive analysis and reporting of the limitations of prediction models serve as an essential foundation for adhering to clinical prediction model guidelines and establishing model transparency and credibility (2). One of the core challenges faced by clinical prediction models lies in ensuring that their development and reporting strictly comply with established clinical prediction model guidelines, which is the primary prerequisite for realizing their clinical translation value.

However, in this study, the reporting of the model’s limitations may not be comprehensive. The authors focused only on the inherent shortcomings of deep learning models, specifically their lack of interpretability due to the “black box” nature, while overlooking other important issues that should be addressed in model interpretation. For example, a key and common limitation of deep learning models is their sensitivity to noise and outliers (3). In real and complex clinical environments, the iShape model could be affected by various sources of “noise,” including technical parameters of equipment, biological and phenotypic heterogeneity across populations, and differences in the standardization of clinical procedures. This sensitivity to noise, if not adequately accounted for, may lead to serious clinical consequences. For instance, in breast cancer diagnosis, it could result in misjudgment of lymph node metastasis status, thereby increasing the risk of inappropriately omitting necessary axillary surgery. These noise factors are directly related to whether the model can be reliably applied in actual clinical practice. Although the model demonstrated excellent performance in the study, its methodology lacks sufficient discussion and analysis of how these potential noise sources are addressed.

Therefore, we strongly recommend that researchers developing and reporting machine learning prediction models in the biomedical field strictly adhere to relevant guidelines (2, 4) to achieve standardization in the reporting of clinical prediction models. In particular, it is essential to provide objective reporting and analysis of the clinical significance and limitations of prediction models, as this is key to establishing reliable clinical prediction models.

Secondly, on the basis of standardized reporting for clinical prediction models, only by further improving the generalization ability of the models and systematically evaluating their inherent limitations can related research truly possess clinical guiding significance. This requires the implementation of a rigorous validation process before a model is put into clinical use: continuously selecting the optimal model, conducting long-term testing of its robustness and generalization ability, and ultimately validating its effectiveness in real-world clinical settings (5). Therefore, the generalization ability and stability of a model are decisive factors in achieving clinical translation.

However, the generalization capability and stability of a model depend on several key factors, including its applicability, sample size (6), and population heterogeneity. In this study, the authors did not thoroughly explore the potential impact of sample size and cohort heterogeneity on the performance of the iShape model. For example, the study trained the model using data from only 371 patients, which may be an insufficient sample size and could affect its stability in real−world applications. Furthermore, in terms of population representativeness, while the training data integrated information from two hospitals in northern and southern regions, the external validation was conducted solely on data from three hospitals in the south. This results in inadequate diversity in geography and demographics in the external validation cohort, which may weaken the model’s generalizability. Although the authors mentioned in the limitations section that large−scale prospective studies are needed in the future, how to systematically address the above heterogeneity challenges remains a critical practical difficulty in implementation.

Therefore, we recommend that future studies should build sufficiently large and adequately diversified training datasets based on clearer clinical objectives, in order to meet the basic data requirements of deep learning. The datasets should strive to cover geographical and demographic variations within the target population, and incorporate variability from different levels of medical institutions and different models of equipment. This approach can help control key confounding factors at the source and ultimately enhance the model’s generalizability and stability. Moreover, external validation should be emphasized, with a focus on evaluating the model’s applicability across diverse populations, real-world clinical settings, and in samples of sufficient size (7–9).

In conclusion, systematic shortcomings in reporting and insufficient robustness are prevalent issues in current clinical prediction model research. Yet, the clinical environment in real-world studies is complex and ever-changing. Consequently, translating prediction models into clinical practice must focus critically on two essential aspects: “standardized reporting” and “rigorous validation.” “Standardized reporting” necessitates addressing all potential limitations of a model, including its algorithmic characteristics and sensitivity to data noise. Meanwhile, “rigorous validation” requires controlling for key confounding factors and overcoming practical constraints. These include data access limitations, multi-center data harmonization, and the execution of prospective validation studies, all of which necessitate long-term and repeated performance assessments of the model. Therefore, future research should adhere to clinical prediction model development guidelines and prioritize establishing a systematic evaluation framework that integrates both “standardized reporting” and “rigorous validation.” This is vital for effectively translating developed prediction models from methodology into clinical practice, transforming them into reliable and practical tools for clinical decision support (10).
